# Expression of *PAX2* and *PAX8* in Wilms Tumor: A Tissue Microarray-based Immunohistochemical Study

**DOI:** 10.30699/IJP.2021.139752.2527

**Published:** 2021-05-09

**Authors:** Salma Sefidbakht, Atieh Khorsand, Sahar Omidi, Sedigheh Mohsenpourian, Elham Mirzaian

**Affiliations:** Department of Pathology, School of Medicine, Tehran University of Medical Sciences, Tehran, Iran

## Abstract

**Background & Objective:**

There is currently inadequate information about the expression of immunohistochemical markers in pediatric tumors. Paired box genes 2 and 8 (*PAX2* and *PAX8*) genes have an essential role in kidney organogenesis. This study aimed to investigate the IHC expression of *PAX2* and *PAX8* in Wilms tumor. Such study would be helpful in diagnosis and possibly in differentiation of this tumor from other mimics, especially in those of poorly differentiated type in small needle biopsy specimens.

**Methods:**

We performed a cross-sectional study on 45 Wilms tumor cases referred to Bahrami pediatric hospital between 2005 and 2015. Demographic data were collected from medical documents. Sections from related paraffin blocks were provided by the tissue microarray method, and immunohistochemical (IHC) staining was done for *PAX8* and *PAX2*.

**Results:**

The mean tumor size was 9.98±4.95 cm. Favorable histology was seen in 84.4% of samples. *PAX2* was expressed in 41 cases (91.1%), and *PAX8* in 37 patients (82.2%). *PAX2* and *PAX8* expression was mostly seen in both blastemal and epithelial components (77.8% and 66.6%), respectively. Tumors with favorable and unfavorable histology did not significantly differ in *PAX2* and *PAX8* expression (*P*=0.637). We found a statically significant relationship between *PAX8* expression and tumor size (*P*=0.033).

**Conclusion:**

*PAX2* and *PAX8* markers might helpful in diagnosis of Wilms tumor and may differentiate it from other histologically similar kidney tumors. *PAX8* expression may be associated with larger tumor size. Tumors with favorable and unfavorable histology may not be different in *PAX2* and *PAX8* expression.

## Introduction

Wilms tumor is the most common abdominal malignancy and kidney tumor in children (1). Since the imaging modalities' diagnostic accuracy is up to 95% in unilateral Wilms tumors and up to 93% in bilateral cases, definitive diagnosis is only possible when tissue examination is done (2). For earlier and more accurate diagnoses, recent years' studies proposed some laboratory and even genetic markers to identify Wilms tumor in children. Previous studies had examined the role of tumor markers in the diagnosis, treatment, and prognosis of other urinary tract tumors such as prostate-specific antigen (PSA) in prostate cancer (3) and chemokine receptor expression, including CXCR3 and CXCR2 in renal cell carcinoma (4).

The paired box genes 2 and 8 (*PAX2* and *PAX8* genes) are members of the paired box (PAX) gene family (5) located on chromosome 10 and 2, respectively (6). They are transcriptional factors that play essential roles in kidney organogenesis (7-9). Both factors are expressed in the Wolffian ducts (10-12), the pronephros' progenitor tissue, and the ureteral bud (12, 13). Each of the *PAX2* or *PAX8* genes are solely sufficient for the formation of pronephros (12). *PAX2* appears to play a more critical role in the formation of mesonephros and metanephros than *PAX8* (2, 12, 13). These markers have been detected in epithelial neoplasms arising in renal and ovarian tissues (5). *PAX2* mutation is associated with autosomal dominant renal coloboma syndrome characterized by congenital anomalies of the kidney, including renal hypoplasia, unilateral agenesis, multicystic dysplastic kidneys, etc. (14). Evaluation of *PAX2* and *PAX8* expression may play a role in detecting abnormalities resulting from the kidney and developmental disorders of the urinary tract. Some studies have demonstrated the presence of both markers in Wilms tumors, renal cell carcinoma, and nephrogenic adenomas (15, 16).

Furthermore, these markers have been introduced as tools to distinguish between benign and malignant tumors of the renal origin (17, 18). However, the expression of these genes in some tumors, such as Wilms, has been less studied. In Iran, no study has been done on the expression of these markers in pediatric tumors, especially Wilms's tumor.

This study aimed to evaluate the expression of *PAX2* and *PAX8* in Wilms tumor using immunohistochemical methods (IHC). It might be helpful in diagnosis of Wilms tumor and differentiation from other histologically similar kidney tumors, especially in needle biopsy specimens.

## Material and Methods

In this cross-sectional study, 45 cases of Wilms tumors were evaluated. The tumors were related to patients who referred to Bahrami pediatric Hospital between 2005 and 2015 and underwent radical nephrectomy. The diagnosis of Wilms tumor in these patients was based on histomorphologic and imaging findings. Information about age, gender, and tumor size were collected from medical documents.

Two pathologists re-examined all patients’ tumor slides. After diagnosis confirmation, the number, and types of tumor components (blastemal, epithelial, and stromal) were determined. Subsequently, the blocks that contained sufficient tumor tissue and less necrosis were chosen for IHC staining.

The selected sections on the hematoxylin and eosin (H&E) slides were then matched with the corresponding blocks and used for tissue microarray construction. Then, we selected tissue cores with a size of 0.6 mm from donor blocks and inserted them into recipient blocks. Two punches from each tumor were incorporated into two paraffin blocks. Five-microns sections of the TMA blocks were transferred to poly-L-lysine slides and then stained for *PAX2* and *PAX8* following the manufacturer's instructions.

We evaluated *PAX2* and *PAX8* in blastemal, epithelial, and stromal components separately. Only moderate to severe nuclear staining was scored as positive.

Based on previous studies, the expression of *PAX8* in Wilms tumor samples was 97%. Assuming a confidence coefficient of 0.05 and an accuracy limit of 0.05, based on the following formula, the sample size required for this study was estimated to be 45 patients.

$N=\frac{P\times \left(1-P\right)\times Z_{1-\alpha /2}^{2}}{d^{2}}$

N = 0.97 × 0.03 × 3.84 / 0.0025 = 45

The results were presented as mean and standard deviation (mean ± SD) for quantitative variables and percentages for qualitative variables. The t-test and the chi-square test were used to compare quantitative and qualitative variables, respectively. The significance level was considered less than 0.05. SPSS 21 (SPSS Inc., Chicago, IL., USA) was used for statistical data analysis.

## Results

The patients' mean age was 35.05±22.90 months. Eighteen children (40%) were male, and 27 children (60%) were female.

Tumor size in these patients was calculated based on the maximum diameter in the specimen's gross examination. The patients' mean tumor size was 9.98±4.95 cm.

On histomorphologic evaluation, 35 cases (77.8%) were triphasic, nine cases (20%) were biphasic, and one case (2.2%) was monophasic. As we evaluated anaplasia as a marker of unfavorable histology, we came to this conclusion. Three cases (6.7%) showed diffuse anaplasia, four cases (8.9%) showed focal anaplasia, and others (84.4%) with no anaplasia.

**Fig. 1:**
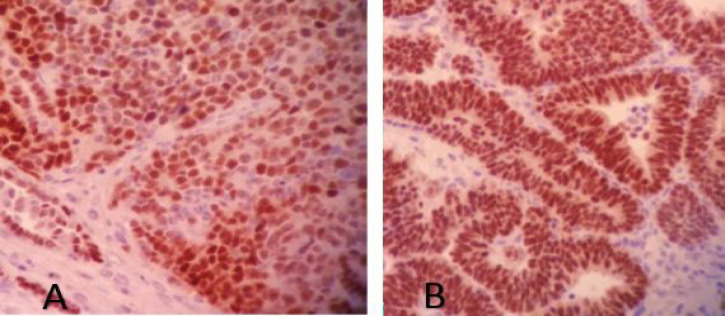
PAX2 immunostaining in: A) blastemal component and B) epithelial component

**Fig. 2:**
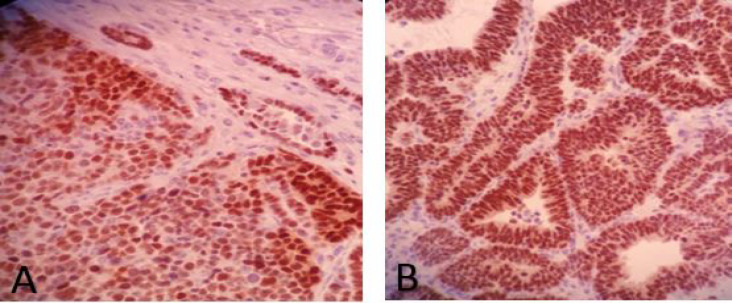
PAX8 immunostaining in: A) blastemal component and B) epithelial component

IHC study showed *PAX2* positivity in 41 cases (91.1%) and *PAX8* positivity in 37 cases (82.2%) (Figures 1 and 2). The expression of these two markers in separate tumor components is shown in Table 1.

We evaluated demographic data and tumor characteristics regarding *PAX2* and *PAX8* expression (Table 2). The only statistically significant relationship was found between *PAX8* expression and tumor size (*P*=0.033). *PAX8* positive tumors demonstrated larger sizes than the others. *PAX2* and *PAX8* expressions were mostly positive in both blastemal and epithelial components (77.8% and 66.6%, respectively). Tumors with favorable and unfavorable histology did not show any significant differences in *PAX2* and *PAX8* expression (*P*=0.637).

**Table 1:** PAX2 and PAX8 expression in different components of Wilms tumor

Tumor components	PAX2 positivity	PAX8 positivity
Epithelial	3(6.7%)	2(4.4%)
Blastemal	3(6.7%)	5(11.1%)
Epithelial and Blastemal	35(77.8%)	30(66.6%)
Stromal	0	0
None	4(8.9%)	8(17.8%)

**Table 2:** PAX2 and PAX8 expression in comparison with demographic data and tumor characteristics

	PAX2			PAX8		
	Positive	Negative	P-value	Positive	Negative	P-value
Age	37.49±23.66	36±0	0.989	36.10±24.03	30.18±17.10	0.514
Gender	M=14 F=27	M=3 F=1	0.672	M=16 F=21	M=2 F=6	0.340
Tumor size	10.48±5.16	10.25±1.72	0.467	10.55±5.12	7.37±3.05	0.033
Histology						
Triphasic Biphasic Monophasic	35 4 2	2 2 0	0.075	29 7 1	6 2 0	0.841
Favorable Unfavorable Focal anaplasia	34 3 4	4 0 0	0.575	31 2 4	7 1 0	0.637

## Discussion

Wilms tumor is the most common kidney tumor in children (2). Nowadays, immunohistochemical markers in various tumors have been widely evaluated, and their use is increasingly expanded for various diagnostic, prognostic, and even therapeutic applications. Some studies have indicated the expression of the *PAX8* and *PAX2* markers in various renal carcinomas in adults, and these markers were known as useful markers in identifying kidney origin tumors. Unlike adults, kidney tumors have been less studied in children, and there is less information on the expression of immunohistochemical markers in pediatric kidney tumors. In Iran, no study has been conducted on the expression of these markers in pediatric tumors, especially Wilms's tumor.

In our study, we used the tissue microarray method. Hundreds of samples can be examined simultaneously under the same conditions, using this new method (19, 20). It is also cost-effective, time-saving, and reagent-saving (19, 21, 22). The amount of tissue required for specific studies is reduced by preserving tissues for further research. However, this method also has limitations. The tissue examined with this technique is limited and may not represent the entire specimen (19, 23). Therefore, it may be challenging to investigate highly heterogeneous tumors (19, 24, 25).

We tried to select tissue cores from donor blocks that include all three blastemal, epithelial, and stromal components.

The current study showed *PAX2* positivity in 41 cases (91.1%) and *PAX8* positivity in 37 cases (82.2%).

In a study by Arva *et al.*, *PAX2* and *PAX8* were positive in all the Wilms tumors but showed variable reactivity in other renal tumors; therefore, they proposed these two markers as sensitive markers with a limited specificity in Wilms tumor diagnosis (26).

Tagge *et al.* also assessed several PAX family genes using Northern blot in Wilms tumor and other childhood neoplasms. They studied 16 Wilms tumor cases (4 primary cases and 12 heterotransplant cases). All four primary Wilms tumors had expressed *PAX2* and *WT1*, and 3 cases had expressed *PAX8* (27). As in the current study, the results of these studies indicate a high frequency of *PAX2* and *PAX8* expression in Wilms tumor, making these markers sensitive markers for diagnosing this tumor. 

As mentioned, 91.1% and 82.2% of studied cases expressed *PAX2* and *PAX8* markers, respectively, with variable intensity, in both blastemal and epithelial components. However, their expression in epithelial and blastemal components was limited. These results indicate that *PAX2* and *PAX8*, concerning their role in the urinary system and kidney development, can show variable expression in various Wilms tumors and can be positive in various Wilms tumor components.

In the current study, tumors with favorable and unfavorable histology did not significantly differ in *PAX2* and *PAX8* expressions. Unfortunately, there is no previous study in this area on Wilms tumor.

We also evaluated *PAX2* and *PAX8* immunostainings related to age, gender, tumor size, and histomorphologic tumor characteristics. The only statistically significant relationship we found was between *PAX8* expression and tumor size, and the frequency of expression of this marker increased significantly as the tumor size increased. Based on previous studies, a critical factor in Wilms tumor prognosis is tumor size. However, no research is currently available on the relationship between tumor size and *PAX2* and *PAX8* expression.

The mean children's age with Wilms tumor in the current study was about 35 months. Worldwide epidemiologic studies have also found that the average involvement age in Wilms tumor patients is between 3 and 4 years (28). The maximum age in our study was nine years, and it is compatible with most of the other sources, which state that all Wilms tumor cases are usually seen before the age of 10 (29). Wilms' frequency in the current study was 1.5 times higher in girls than boys. The boys’ and the girls’ mean age was 29 months and 38 months, respectively.

Another survey by Hemmatyar *et al.* at Tehran Pediatric Medical Center also found the mean age of 3.5 years, which is slightly higher than the current study. In their study, 66% of the children were girls which is in consistence with our study (30). Worldwide studies have shown that the Wilms tumor prevalence is slightly higher in girls (31). 

One of the critical limitations of the current study was the small sample size. Further studies with a larger sample size are needed to examine the correlation between the expression of these IHC markers and the clinicopathological parameters. We also recommend the investigation of these markers in differential diagnosis of Wilms tumor. 

## Conclusion

This study aimed to investigate the immunohistochemical expression of *PAX2* and *PAX8* in Wilms tumor for diagnosing this tumor and possibly differentiating it from other differentials. These markers are probably useful in differentiating Wilms tumor from poorly differentiated tumors in the small needle biopsy specimens.

The current study demonstrated *PAX2* expression in 91.1% of Wilms tumor cases and *PAX8* expression in 82.2% of cases. *PAX8* expression was associated with larger tumor size. Tumors with favorable and unfavorable histology did not show significant differences in *PAX2* and *PAX8* expression.

## References

[1] Varan A (2008). Wilms' tumor in children: an overview. Nephron Clinical Practice.

[2] Lonergan GJ, Martinez-Leon MI, Agrons GA, Montemarano H, Suarez ES (1998). Nephrogenic rests, nephroblastomatosis, and associated lesions of the kidney. Radiographics.

[3] Frank JD, Gearhart JP, Snyder HM (2002). Operative pediatric urology.

[4] Dressler GR (2006). The cellular basis of kidney development. Annu Rev Cell Dev Biol..

[5] Eccles MR, He S, Legge M, Kumar R, Fox J, Zhou C (2004). PAX genes in development and disease: the role of PAX2 in urogenital tract development. International Journal of Developmental Biology.

[6] Tong G-X, Woojin MY, Beaubier NT, Weeden EM, Hamele-Bena D, Mansukhani MM (2009). Expression of PAX8 in normal and neoplastic renal tissues: an immunohistochemical study. Modern Pathology.

[7] Ozcan A, De La Roza G, Ro JY, Shen SS, Truong LD (2012). PAX2 and PAX8 expression in primary and metastatic renal tumors: a comprehensive comparison. Archives of pathology & laboratory medicine.

[8] Bouchard M, Souabni A, Mandler M, Neubüser A, Busslinger M (2002). Nephric lineage specification by Pax2 and Pax8. Genes & development.

[9] Narlis M, Grote D, Gaitan Y, Boualia SK, Bouchard M (2007). Pax2 and pax8 regulate branching morphogenesis and nephron differentiation in the developing kidney. Journal of the American Society of Nephrology.

[10] Grote D, Souabni A, Busslinger M, Bouchard M (2006). Pax2/8-regulated Gata3 expression is necessary for morphogenesis and guidance of the nephric duct in the developing kidney. Development.

[11] Daniel L, Lechevallier E, Giorgi R, Sichez H, Zattara-Cannoni H, Figarella-Branger D (2001). Pax-2 expression in adult renal tumors. Human pathology.

[12] Eccles M, Wallis L, Fidler A, Spurr N, Goodfellow P, Reeve A (1992). Expression of the PAX2 gene in human fetal kidney and Wilms' tumor. Cell Growth Differ.

[13] Poleev A, Fickenscher H, Mundlos S, Winterpacht A, Zabel B, Fidler A (1992). PAX8, a human paired box gene: isolation and expression in developing thyroid, kidney and Wilms' tumors. Development.

[14] Tong G-X, Weeden EM, Hamele-Bena D, Huan Y, Unger P, Memeo L (2008). Expression of PAX8 in nephrogenic adenoma and clear cell adenocarcinoma of the lower urinary tract: evidence of related histogenesis?. The American journal of surgical pathology.

[15] Tagge EP, Hanson P, Re GG, Othersen Jr HB, Smith CD, Garvin AJ (1994). Paired box gene expression in Wilms' tumor. Journal of pediatric surgery.

[16] Khouja MH, Baekelandt M, Sarab A, Nesland JM, Holm R (2010). Limitations of tissue microarrays compared with whole tissue sections in survival analysis. Oncology letters.

[17] Kononen J, Bubendorf L, Kallionimeni A, Bärlund M, Schraml P, Leighton S (1998). Tissue microarrays for high-throughput molecular profiling of tumor specimens. Nature medicine.

[18] Mills SE, Fechner RE, Frierson HF, Kempson RL, Wick MR, Dehner LP (1995). Guardians of the wax… and the patient. American journal of clinical pathology.

[19] Camp RL, Charette LA, Rimm DL (2000). Validation of tissue microarray technology in breast carcinoma. Laboratory investigation.

[20] Griffin MC, Robinson RA, Trask DK (2003). Validation of tissue microarrays using p53 immunohistochemical studies of squamous cell carcinoma of the larynx. Modern pathology.

[21] Kuwabara S, Ajioka Y, Watanabe H, Hitomi J, Nishikura K, Hatakeyama K (1998). Heterogeneity of p53 mutational status in esophageal squamous cell carcinoma. Japanese journal of cancer research.

[22] Baisse B, Bouzourene H, Saraga EP, Bosman FT, Benhattar J (2001). Intratumor genetic heterogeneity in advanced human colorectal adenocarcinoma. International journal of cancer.

[23] Nonaka D, Tang Y, Chiriboga L, Rivera M, Ghossein R (2008). Diagnostic utility of thyroid transcription factors Pax8 and TTF-2 (FoxE1) in thyroid epithelial neoplasms. Modern Pathology.

[24] Laury AR, Hornick JL, Perets R, Krane JF, Corson J, Drapkin R (2010). PAX8 reliably distinguishes ovarian serous tumors from malignant mesothelioma. The American journal of surgical pathology.

[25] Knoepp SM, Kunju LP, Roh MH (2012). Utility of PAX8 and PAX2 immunohistochemistry in the identification of renal cell carcinoma in diagnostic cytology. Diagnostic cytopathology.

[26] Arva NC, Bonadio J, Perlman EJ, Cajaiba MM (2018). Diagnostic utility of Pax8, Pax2, and NGFR immunohistochemical expression in pediatric renal tumors. Applied immunohistochemistry & molecular morphology.

[27] Verschuur AC, Vujanic GM, Van Tinteren H, Jones KP, de Kraker J, Sandstedt B (2010). Stromal and epithelial predominant Wilms tumours have an excellent outcome: the SIOP 93 01 experience. Pediatric blood & cancer.

[28] Charles A, Vujanic G, Berry P (1998). Renal tumours of childhood. Histopathology.

[29] Hrabovsky EE, Othersen Jr HB, deLorimier A, Kelalis P, Beckwith JB, Takashima J (1986). Wilms' tumor in the neonate: a report from the National Wilms' Tumor Study. Journal of pediatric surgery.

[30] Hemaatyar M, Robat Mili M (2006). Comparison of clinical manifestation, age and sex distribution in childhood Wilms' tumor and neuroblastoma in Tehran Children Medical Center hospital. Medical Science Journal of Islamic Azad Univesity-Tehran Medical Branch.

[31] Breslow N, Olshan A, Beckwith JB, Green DM (1993). Epidemiology of Wilms tumor. Medical and pediatric oncology.

